# Supplementary material for: Pancreatitis Risk Associated with GLP-1 Receptor Agonists, Considered as a Single Class, in a Comorbidity-Free Subgroup of Type 2 Diabetes Patients in the United States: A Propensity Score-Matched Analysis
Source: J Clin Med. 2025 Feb 1;14(3):944. doi: 10.3390/jcm14030944 (PMC11818918; doi:10.3390/jcm14030944)
Supplement: Supplementary file 1 [file jcm-14-00944-s001.zip › jcm-3392393-supplementary.pdf]

Table S1. List of all components used in PSM.

Demographics	Comorbidities	Medications				Labs
1. Age at Index<br>2. White<br>3. Unknown Race<br>4. Female<br>5. Unknown Ethnicity<br>6. Hispanic or Latino<br>7. Not Hispanic or Latino<br>8. Black or African American<br>9. Male<br>10. Asian	1. Endocrine, nutritional and metabolic diseases<br>2. Hypertensive diseases<br>3. Ischemic heart diseases<br>4. Calculus of gallbladder without cholecystitis without obstruction<br>5. Annular pancreas<br>6. Alcohol related disorders<br>7. Pure hyperglyceridemia<br>8. Hypercalcemia<br>9. Cystic fibrosis<br>10. Cannabis related disorders	1. pantoprazole<br>2. omeprazole<br>3. dexlansoprazole<br>4. linagliptin<br>5. alogliptin<br>6. sitagliptin<br>7. saxagliptin<br>8. rosuvastatin<br>9. simvastatin<br>10. fluvastatin<br>11. pravastatin<br>12. atorvastatin<br>13. pitavastatin<br>14. vincristine<br>15. voriconazole<br>16. zolmitriptan<br>17. ADRENAL CORTICOSTEROIDS	18. prazosin<br>19. procainamide<br>20. propofol<br>21. isotretinoin<br>22. acetaminophen<br>23. metformin<br>24. methimazole<br>25. dipyridamole<br>26. diphenoxylate<br>27. amlodipine<br>28. ampicillin<br>29. aspirin<br>30. ANTIVIRALS<br>31. sumatriptan<br>32. tacrolimus<br>33. tamoxifen<br>34. TETRACYCLINES	35. octreotide<br>36. PENICILLINS AND BETA-LACTAM ANTIMICROBIALS<br>37. pentamidine<br>38. pilocarpine<br>39. doxorubicin<br>40. ertapenem<br>41. ESTROGENS<br>42. ANTIPSYCHOTICS<br>43. azathioprine<br>44. bupropion<br>45. CALCITRIOL<br>46. carbamazepine<br>47. ceftriaxone<br>48. cimetidine<br>49. cisplatin<br>50. clomiphene<br>51. codeine	52. NON-STEROIDAL ANTI-INFLAMMATORY ANALGESICS<br>53. alendronate<br>54. paroxetine<br>55. sertraline<br>56. sirolimus<br>57. doxercalciferol<br>58. irbesartan<br>59. cyclophosphamide<br>60. citalopram<br>61. escitalopram<br>62. THIAZIDES/RELATED DIURETICS<br>63. TUMOR NECROSIS FACTOR BLOCKER<br>64. topiramate<br>65. valproate<br>66. venlafaxine<br>67. colchicine	1. Hemoglobin A1c/Hemoglobin.total in Blood<br>2. BMI<br>3. Triglyceride [Mass/volume] in Serum, Plasma or Blood<br>4. Calcium [Mass/volume] in Serum, Plasma or Blood

Table S2. Cohorts, patient count, and PSM components before and after matching.

Cohorts and patient count before and after propensity score matching		
Cohort	Patient count before matching	Patient count after matching
GLP-1 RA	93,608	82,333
No GLP-1 RA	875,632	82,333

Propensity score density function - Before and after matching (GLP-1 RA - purple, No GLP-1 RA - green)

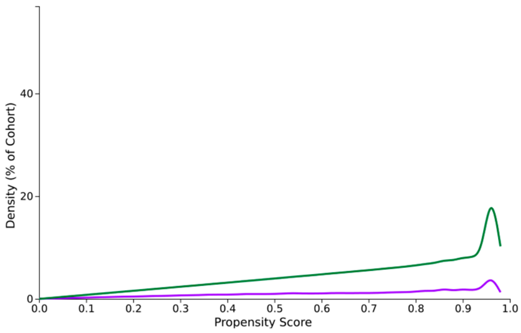

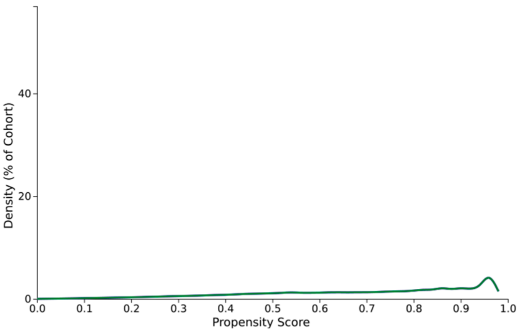

GLP-1 RA (N = 93,608) and No GLP-1 RA (N = 875,632) characteristics before propensity score matching

Demographics

Cohort		Mean ± SD	Patients	% of Cohort	P-Value
GLP-1 RA<br>No GLP-1 RA	Age at Index	47.5 +/- 11.8<br>46.3 +/- 12.7	93,608<br>862,289	100%<br>100%	<0.001
GLP-1 RA<br>No GLP-1 RA	White		55,482<br>455,059	59.3%<br>52.8%	<0.001
GLP-1 RA<br>No GLP-1 RA	Unknown Race		13,020<br>170,450	13.9%<br>19.8%	<0.001
GLP-1 RA<br>No GLP-1 RA	Female		59,686<br>455,982	63.8%<br>52.9%	<0.001
GLP-1 RA<br>No GLP-1 RA	Unknown Ethnicity		25,735<br>290,251	27.5%<br>33.7%	<0.001
GLP-1 RA<br>No GLP-1 RA	Hispanic or Latino		11,807<br>141,163	12.6%<br>16.4%	<0.001
GLP-1 RA<br>No GLP-1 RA	Not Hispanic or Latino		56,066<br>430,875	59.9%<br>50.0%	<0.001
GLP-1 RA<br>No GLP-1 RA	Black or African American		15,226<br>131,026	16.3%<br>15.2%	<0.001
GLP-1 RA<br>No GLP-1 RA	Male		30,901<br>382,507	33.0%<br>44.4%	<0.001
GLP-1 RA<br>No GLP-1 RA	Asian		3,390<br>41,088	3.6%<br>4.8%	<0.001

Diagnosis

Cohort			Patients	% of Cohort	P-Value
GLP-1 RA<br>No GLP-1 RA	E00-E89	Endocrine, nutritional and metabolic diseases	70,700<br>171,261	75.5%<br>19.9%	<0.001
GLP-1 RA<br>No GLP-1 RA	K80.20	Calculus of gallbladder without cholecystitis without obstruction	2,222<br>8,773	2.4%<br>1.0%	<0.001
GLP-1 RA<br>No GLP-1 RA	Q45.1	Annular pancreas	10<br>68	0.0%<br>0.0%	<0.001
GLP-1 RA<br>No GLP-1 RA	F10	Alcohol related disorders	1,304<br>10,907	1.4%<br>1.3%	0.001
GLP-1 RA<br>No GLP-1 RA	E78.1	Pure hyperglyceridemia	3,428<br>6,076	3.7%<br>0.7%	<0.001
GLP-1 RA<br>No GLP-1 RA	E83.52	Hypercalcemia	690<br>1,682	0.7%<br>0.2%	<0.001
GLP-1 RA<br>No GLP-1 RA	E84	Cystic fibrosis	33<br>1,286	0.0%<br>0.1%	<0.001
GLP-1 RA<br>No GLP-1 RA	F12	Cannabis related disorders	822<br>5,797	0.9%<br>0.7%	<0.001

Medication

Cohort		Patients	% of Cohort	P-Value
GLP-1 RA<br>No GLP-1 RA	pantoprazole	9,843<br>31,993	10.5%<br>3.7%	<0.001
GLP-1 RA<br>No GLP-1 RA	omeprazole	13,577<br>38,183	14.5%<br>4.4%	<0.001
GLP-1 RA<br>No GLP-1 RA	dexlansoprazole	588<br>1,370	0.6%<br>0.2%	<0.001
GLP-1 RA<br>No GLP-1 RA	linagliptin	1,808<br>1,326	1.9%<br>0.2%	<0.001
GLP-1 RA<br>No GLP-1 RA	alogliptin	486<br>326	0.5%<br>0.0%	<0.001
GLP-1 RA<br>No GLP-1 RA	sitagliptin	9,315<br>8,094	10.0%<br>0.9%	<0.001
GLP-1 RA<br>No GLP-1 RA	saxagliptin	617<br>594	0.7%<br>0.1%	<0.001

GLP-1 RA<br>No GLP-1 RA	rosuvastatin	7,497<br>10,635	8.0%<br>1.2%	<0.001
GLP-1 RA<br>No GLP-1 RA	simvastatin	5,574<br>13,651	6.0%<br>1.6%	<0.001
GLP-1 RA<br>No GLP-1 RA	fluvastatin	36<br>53	0.0%<br>0.0%	<0.001
GLP-1 RA<br>No GLP-1 RA	pravastatin	3,558<br>6,726	3.8%<br>0.8%	<0.001
GLP-1 RA<br>No GLP-1 RA	atorvastatin	22,322<br>35,757	23.8%<br>4.1%	<0.001
GLP-1 RA<br>No GLP-1 RA	pitavastatin	130<br>210	0.1%<br>0.0%	<0.001
GLP-1 RA<br>No GLP-1 RA	NON-STEROIDAL ANTI-INFLAMMATORY ANALGESICS	23,130<br>92,723	24.7%<br>10.8%	<0.001
GLP-1 RA<br>No GLP-1 RA	alendronate	359<br>1,804	0.4%<br>0.2%	<0.001
GLP-1 RA<br>No GLP-1 RA	octreotide	82<br>1,023	0.1%<br>0.1%	0.008
GLP-1 RA<br>No GLP-1 RA	PENICILLINS AND BETA-LACTAM ANTIMICROBIALS	33,308<br>121,576	35.6%<br>14.1%	<0.001
GLP-1 RA<br>No GLP-1 RA	pentamidine	21<br>174	0.0%<br>0.0%	<0.001
GLP-1 RA<br>No GLP-1 RA	pilocarpine	169<br>624	0.2%<br>0.1%	<0.001
GLP-1 RA<br>No GLP-1 RA	prazosin	740<br>2,062	0.8%<br>0.2%	<0.001
GLP-1 RA<br>No GLP-1 RA	procainamide	10<br>16	0.0%<br>0.0%	<0.001
GLP-1 RA<br>No GLP-1 RA	propofol	16,202<br>47,479	17.3%<br>5.5%	<0.001
GLP-1 RA<br>No GLP-1 RA	isotretinoin	113<br>276	0.1%<br>0.0%	<0.001
GLP-1 RA<br>No GLP-1 RA	acetaminophen	35,331<br>150,376	37.7%<br>17.4%	<0.001
GLP-1 RA<br>No GLP-1 RA	metformin	55,102<br>92,892	58.9%<br>10.8%	<0.001
GLP-1 RA<br>No GLP-1 RA	methimazole	367<br>1,153	0.4%<br>0.1%	<0.001
GLP-1 RA<br>No GLP-1 RA	dipyridamole	54<br>173	0.1%<br>0.0%	<0.001
GLP-1 RA<br>No GLP-1 RA	diphenoxylate	966<br>3,711	1.0%<br>0.4%	<0.001
GLP-1 RA<br>No GLP-1 RA	amlodipine	2,284<br>7,560	2.4%<br>0.9%	<0.001
GLP-1 RA<br>No GLP-1 RA	ampicillin	1,189<br>5,562	1.3%<br>0.6%	<0.001
GLP-1 RA<br>No GLP-1 RA	aspirin	11,255<br>34,708	12.0%<br>4.0%	<0.001
GLP-1 RA<br>No GLP-1 RA	ANTIVIRALS	10,791<br>26,693	11.5%<br>3.1%	<0.001
GLP-1 RA<br>No GLP-1 RA	sumatriptan	3,459<br>7,547	3.7%<br>0.9%	<0.001
GLP-1 RA<br>No GLP-1 RA	tacrolimus	592<br>1,972	0.6%<br>0.2%	<0.001

GLP-1 RA<br>No GLP-1 RA	tamoxifen	348<br>967	0.4%<br>0.1%	<0.001
GLP-1 RA<br>No GLP-1 RA	TETRACYCLINES	11,480<br>28,593	12.3%<br>3.3%	<0.001
GLP-1 RA<br>No GLP-1 RA	THIAZIDES/RELATED DIURETICS	4,726<br>12,343	5.0%<br>1.4%	<0.001
GLP-1 RA<br>No GLP-1 RA	TUMOR NECROSIS FACTOR BLOCKER	53<br>124	0.1%<br>0.0%	<0.001
GLP-1 RA<br>No GLP-1 RA	topiramate	4,966<br>8,697	5.3%<br>1.0%	<0.001
GLP-1 RA<br>No GLP-1 RA	valproate	929<br>5,291	1.0%<br>0.6%	<0.001
GLP-1 RA<br>No GLP-1 RA	venlafaxine	3,731<br>8,403	4.0%<br>1.0%	<0.001
GLP-1 RA<br>No GLP-1 RA	vincristine	27<br>371	0.0%<br>0.0%	0.043
GLP-1 RA<br>No GLP-1 RA	voriconazole	33<br>487	0.0%<br>0.1%	0.008
GLP-1 RA<br>No GLP-1 RA	zolmitriptan	245<br>558	0.3%<br>0.1%	<0.001
GLP-1 RA<br>No GLP-1 RA	ADRENAL CORTICOSTEROIDS	39,952<br>135,782	42.7%<br>15.7%	<0.001
GLP-1 RA<br>No GLP-1 RA	citalopram	3,793<br>10,383	4.1%<br>1.2%	<0.001
GLP-1 RA<br>No GLP-1 RA	escitalopram	6,822<br>13,889	7.3%<br>1.6%	<0.001
GLP-1 RA<br>No GLP-1 RA	paroxetine	1,659<br>4,901	1.8%<br>0.6%	<0.001
GLP-1 RA<br>No GLP-1 RA	sertraline	7,121<br>17,710	7.6%<br>2.1%	<0.001
GLP-1 RA<br>No GLP-1 RA	sirolimus	10<br>121	0.0%<br>0.0%	<0.001
GLP-1 RA<br>No GLP-1 RA	doxercalciferol	0<br>10	0%<br>0.0%	<0.001
GLP-1 RA<br>No GLP-1 RA	doxorubicin	141<br>826	0.2%<br>0.1%	<0.001
GLP-1 RA<br>No GLP-1 RA	ertapenem	246<br>1,337	0.3%<br>0.2%	<0.001
GLP-1 RA<br>No GLP-1 RA	ESTROGENS	4,045<br>9,414	4.3%<br>1.1%	<0.001
GLP-1 RA<br>No GLP-1 RA	ANTIPSYCHOTICS	7,683<br>30,565	8.2%<br>3.5%	<0.001
GLP-1 RA<br>No GLP-1 RA	azathioprine	269<br>1,031	0.3%<br>0.1%	<0.001
GLP-1 RA<br>No GLP-1 RA	bupropion	8,324<br>14,506	8.9%<br>1.7%	<0.001
GLP-1 RA<br>No GLP-1 RA	CALCITRIOL	180<br>712	0.2%<br>0.1%	<0.001
GLP-1 RA<br>No GLP-1 RA	carbamazepine	411<br>1,800	0.4%<br>0.2%	<0.001
GLP-1 RA<br>No GLP-1 RA	ceftriaxone	4,101<br>17,070	4.4%<br>2.0%	<0.001
GLP-1 RA<br>No GLP-1 RA	cimetidine	152<br>494	0.2%<br>0.1%	<0.001
GLP-1 RA<br>No GLP-1 RA	cisplatin	47<br>480	0.1%<br>0.1%	<0.001

GLP-1 RA<br>No GLP-1 RA	clomiphene	496<br>1,394	0.5%<br>0.2%	<0.001
GLP-1 RA<br>No GLP-1 RA	codeine	9,608<br>29,690	10.3%<br>3.4%	<0.001
GLP-1 RA<br>No GLP-1 RA	colchicine	722<br>2,163	0.8%<br>0.3%	<0.001
GLP-1 RA<br>No GLP-1 RA	irbesartan	275<br>459	0.3%<br>0.1%	<0.001
GLP-1 RA<br>No GLP-1 RA	cyclophosphamide	165<br>970	0.2%<br>0.1%	<0.001

Laboratory

Cohort		Mean ± SD	Patients	% of Cohort	P-Value
GLP-1 RA<br>No GLP-1 RA	Hemoglobin A1c total in Blood	8.1 +/- 2.2<br>7.3 +/- 2.2	55,034<br>151,582	58.8%<br>17.6%	<0.001
GLP-1 RA<br>No GLP-1 RA	BMI	35.5 +/- 6.9<br>32.4 +/- 7.3	24,572<br>120,183	26.2%<br>13.9%	<0.001
GLP-1 RA<br>No GLP-1 RA	Triglyceride in Serum, Plasma or Blood	187.0 +/- 171.2<br>180.8 +/- 214.0	49,446<br>133,471	52.8%<br>15.5%	<0.001
GLP-1 RA<br>No GLP-1 RA	Calcium in Serum, Plasma or Blood	9.4 +/- 0.5<br>9.3 +/- 0.6	60,900<br>240,879	65.1%<br>27.9%	<0.001

GLP-1 RA (N = 82,333) and No GLP-1 RA (N = 82,333) characteristics after propensity score matching

Demographics

Cohort		Mean ± SD	Patients	% of Cohort	P-Value
GLP-1 RA<br>No GLP-1 RA	Age at Index	47.3 +/- 11.9<br>47.9 +/- 12.5	82,333<br>82,333	100%<br>100%	0.275
GLP-1 RA<br>No GLP-1 RA	White		47,983<br>48,794	58.3%<br>59.3%	0.873
GLP-1 RA<br>No GLP-1 RA	Unknown Race		11,975<br>11,429	14.5%<br>13.9%	0.624
GLP-1 RA<br>No GLP-1 RA	Female		51,568<br>51,607	62.6%<br>62.7%	0.843
GLP-1 RA<br>No GLP-1 RA	Unknown Ethnicity		22,992<br>22,124	27.9%<br>26.9%	0.287
GLP-1 RA<br>No GLP-1 RA	Hispanic or Latino		10,790<br>9,971	13.1%<br>12.1%	0.312
GLP-1 RA<br>No GLP-1 RA	Not Hispanic or Latino		48,551<br>50,238	59.0%<br>61.0%	0.292
GLP-1 RA<br>No GLP-1 RA	Black or African American		13,461<br>13,295	16.3%<br>16.1%	0.267
GLP-1 RA<br>No GLP-1 RA	Male		28,193<br>28,305	34.2%<br>34.4%	0.561
GLP-1 RA<br>No GLP-1 RA	Asian		3,109<br>3,198	3.8%<br>3.9%	0.253

Diagnosis

Cohort			Patients	% of Cohort	P-Value
GLP-1 RA<br>No GLP-1 RA	E00-E89	Endocrine, nutritional and metabolic diseases	60,331<br>34,772	73.3%<br>42.2%	0.065
GLP-1 RA<br>No GLP-1 RA	K80.20	Calculus of gallbladder without cholecystitis without obstruction	1,724<br>1,634	2.1%<br>2.0%	0.117
GLP-1 RA<br>No GLP-1 RA	Q45.1	Annular pancreas	10<br>18	0.0%<br>0.0%	0.131

GLP-1 RA<br>No GLP-1 RA	F10	Alcohol related disorders	1,165<br>1,118	1.4%<br>1.4%	0.322
GLP-1 RA<br>No GLP-1 RA	E78.1	Pure hyperglyceridemia	2,639<br>1,683	3.2%<br>2.0%	0.092
GLP-1 RA<br>No GLP-1 RA	E83.52	Hypercalcemia	508<br>433	0.6%<br>0.5%	0.065
GLP-1 RA<br>No GLP-1 RA	E84	Cystic fibrosis	32<br>57	0.0%<br>0.1%	0.082
GLP-1 RA<br>No GLP-1 RA	F12	Cannabis related disorders	716<br>666	0.9%<br>0.8%	0.177

Medication

Cohort		Patients	% of Cohort	P-Value
GLP-1 RA<br>No GLP-1 RA	pantoprazole	7,866<br>8,035	9.6%<br>9.8%	0.159
GLP-1 RA<br>No GLP-1 RA	omeprazole	10,601<br>10,731	12.9%<br>13.0%	0.340
GLP-1 RA<br>No GLP-1 RA	dexlansoprazole	460<br>453	0.6%<br>0.6%	0.816
GLP-1 RA<br>No GLP-1 RA	linagliptin	920<br>838	1.1%<br>1.0%	0.049
GLP-1 RA<br>No GLP-1 RA	alogliptin	224<br>208	0.3%<br>0.3%	0.441
GLP-1 RA<br>No GLP-1 RA	sitagliptin	5,307<br>5,060	6.4%<br>6.1%	0.072
GLP-1 RA<br>No GLP-1 RA	saxagliptin	372<br>347	0.5%<br>0.4%	0.350
GLP-1 RA<br>No GLP-1 RA	rosuvastatin	5,199<br>5,262	6.3%<br>6.4%	0.524
GLP-1 RA<br>No GLP-1 RA	simvastatin	4,385<br>4,505	5.3%<br>5.5%	0.191
GLP-1 RA<br>No GLP-1 RA	fluvastatin	24<br>20	0.0%<br>0.0%	0.546
GLP-1 RA<br>No GLP-1 RA	pravastatin	2,644<br>2,645	3.2%<br>3.2%	0.989
GLP-1 RA<br>No GLP-1 RA	atorvastatin	16,181<br>16,405	19.7%<br>19.9%	0.166
GLP-1 RA<br>No GLP-1 RA	pitavastatin	99<br>88	0.1%<br>0.1%	0.421
GLP-1 RA<br>No GLP-1 RA	NON-STEROIDAL ANTI-INFLAMMATORY ANALGESICS	18,865<br>18,860	22.9%<br>22.9%	0.072
GLP-1 RA<br>No GLP-1 RA	alendronate	310<br>350	0.4%<br>0.4%	0.119
GLP-1 RA<br>No GLP-1 RA	octreotide	79<br>74	0.1%<br>0.1%	0.686
GLP-1 RA<br>No GLP-1 RA	PENICILLINS AND BETA-LACTAM ANTIMICROBIALS	26,732<br>26,996	32.5%<br>32.8%	0.069
GLP-1 RA<br>No GLP-1 RA	pentamidine	19<br>25	0.0%<br>0.0%	0.366
GLP-1 RA<br>No GLP-1 RA	pilocarpine	143<br>156	0.2%<br>0.2%	0.452
GLP-1 RA<br>No GLP-1 RA	prazosin	580<br>583	0.7%<br>0.7%	0.930
GLP-1 RA<br>No GLP-1 RA	procainamide	10<br>10	0.0%<br>0.0%	1
GLP-1 RA<br>No GLP-1 RA	propofol	12,342<br>12,367	15.0%<br>15.0%	0.863

GLP-1 RA<br>No GLP-1 RA	isotretinoin	88<br>88	0.1%<br>0.1%	1
GLP-1 RA<br>No GLP-1 RA	acetaminophen	29,208<br>29,848	35.5%<br>36.3%	0.192
GLP-1 RA<br>No GLP-1 RA	metformin	43,937<br>43,962	53.4%<br>53.4%	0.902
GLP-1 RA<br>No GLP-1 RA	methimazole	303<br>302	0.4%<br>0.4%	0.968
GLP-1 RA<br>No GLP-1 RA	dipyridamole	47<br>49	0.1%<br>0.1%	0.838
GLP-1 RA<br>No GLP-1 RA	diphenoxylate	803<br>811	1.0%<br>1.0%	0.841
GLP-1 RA<br>No GLP-1 RA	amlodipine	2,028<br>2,245	2.5%<br>2.7%	0.091
GLP-1 RA<br>No GLP-1 RA	ampicillin	1,010<br>1,005	1.2%<br>1.2%	0.911
GLP-1 RA<br>No GLP-1 RA	aspirin	9,059<br>9,248	11.0%<br>11.2%	0.138
GLP-1 RA<br>No GLP-1 RA	ANTIVIRALS	8,018<br>8,038	9.7%<br>9.8%	0.868
GLP-1 RA<br>No GLP-1 RA	sumatriptan	2,567<br>2,599	3.1%<br>3.2%	0.651
GLP-1 RA<br>No GLP-1 RA	tacrolimus	467<br>484	0.6%<br>0.6%	0.580
GLP-1 RA<br>No GLP-1 RA	tamoxifen	260<br>281	0.3%<br>0.3%	0.366
GLP-1 RA<br>No GLP-1 RA	TETRACYCLINES	8,541<br>8,518	10.4%<br>10.3%	0.852
GLP-1 RA<br>No GLP-1 RA	THIAZIDES/RELATED DIURETICS	3,939<br>4,186	4.8%<br>5.1%	0.076
GLP-1 RA<br>No GLP-1 RA	TUMOR NECROSIS FACTOR BLOCKER	41<br>33	0.0%<br>0.0%	0.059
GLP-1 RA<br>No GLP-1 RA	topiramate	3,498<br>3,532	4.2%<br>4.3%	0.679
GLP-1 RA<br>No GLP-1 RA	valproate	828<br>836	1.0%<br>1.0%	0.844
GLP-1 RA<br>No GLP-1 RA	venlafaxine	2,765<br>2,753	3.4%<br>3.3%	0.869
GLP-1 RA<br>No GLP-1 RA	vincristine	27<br>32	0.0%<br>0.0%	0.515
GLP-1 RA<br>No GLP-1 RA	voriconazole	32<br>46	0.0%<br>0.1%	0.113
GLP-1 RA<br>No GLP-1 RA	zolmitriptan	183<br>172	0.2%<br>0.2%	0.559
GLP-1 RA<br>No GLP-1 RA	ADRENAL CORTICOSTEROIDS	32,031<br>32,663	38.9%<br>39.7%	0.892
GLP-1 RA<br>No GLP-1 RA	citalopram	2,901<br>2,951	3.5%<br>3.6%	0.506
GLP-1 RA<br>No GLP-1 RA	escitalopram	4,998<br>5,028	6.1%<br>6.1%	0.757
GLP-1 RA<br>No GLP-1 RA	paroxetine	1,337<br>1,352	1.6%<br>1.6%	0.771
GLP-1 RA<br>No GLP-1 RA	sertraline	5,401<br>5,441	6.6%<br>6.6%	0.691
GLP-1 RA<br>No GLP-1 RA	sirolimus	10<br>22	0.0%<br>0.0%	0.034

GLP-1 RA<br>No GLP-1 RA	doxercalciferol		0<br>10	0%<br>0.0%	0.058
GLP-1 RA<br>No GLP-1 RA	doxorubicin		118<br>115	0.1%<br>0.1%	0.844
GLP-1 RA<br>No GLP-1 RA	ertapenem		212<br>236	0.3%<br>0.3%	0.256
GLP-1 RA<br>No GLP-1 RA	ESTROGENS		3,067<br>3,124	3.7%<br>3.8%	0.460
GLP-1 RA<br>No GLP-1 RA	ANTIPSYCHOTICS		6,368<br>6,558	7.7%<br>8.0%	0.082
GLP-1 RA<br>No GLP-1 RA	azathioprine		231<br>228	0.3%<br>0.3%	0.888
GLP-1 RA<br>No GLP-1 RA	bupropion		5,847<br>5,810	7.1%<br>7.1%	0.722
GLP-1 RA<br>No GLP-1 RA	CALCITRIOL		151<br>162	0.2%<br>0.2%	0.534
GLP-1 RA<br>No GLP-1 RA	carbamazepine		337<br>358	0.4%<br>0.4%	0.425
GLP-1 RA<br>No GLP-1 RA	ceftriaxone		3,332<br>3,283	4.0%<br>4.0%	0.539
GLP-1 RA<br>No GLP-1 RA	cimetidine		124<br>110	0.2%<br>0.1%	0.360
GLP-1 RA<br>No GLP-1 RA	cisplatin		40<br>58	0.0%<br>0.1%	0.069
GLP-1 RA<br>No GLP-1 RA	clomiphene		423<br>434	0.5%<br>0.5%	0.706
GLP-1 RA<br>No GLP-1 RA	codeine		7,430<br>7,450	9.0%<br>9.0%	0.864
GLP-1 RA<br>No GLP-1 RA	colchicine		565<br>548	0.7%<br>0.7%	0.609
GLP-1 RA<br>No GLP-1 RA	irbesartan		199<br>207	0.2%<br>0.3%	0.691
GLP-1 RA<br>No GLP-1 RA	cyclophosphamide		144<br>156	0.2%<br>0.2%	0.488
**Laboratory**					
Cohort		Mean ± SD	Patients	% of Cohort	P-Value
GLP-1 RA<br>No GLP-1 RA	Hemoglobin A1c total in Blood	8.1 +/- 2.3<br>7.2 +/- 2.1	44,297<br>44,306	53.8%<br>53.8%	0.169
GLP-1 RA<br>No GLP-1 RA	BMI	35.6 +/- 6.9<br>33.4 +/- 7.3	21,109<br>21,516	25.6%<br>26.1%	0.096
GLP-1 RA<br>No GLP-1 RA	Triglyceride in Serum, Plasma or Blood	186.9 +/- 174.8<br>178.8 +/- 176.3	39,112<br>38,478	47.5%<br>46.7%	0.275
GLP-1 RA<br>No GLP-1 RA	Calcium in Serum, Plasma or Blood	9.4 +/- 0.5<br>9.3 +/- 0.6	50,125<br>50,451	60.9%<br>61.3%	0.158
